# Nitric Oxide and Hydrogen Peroxide Signaling in Extractive *Shiraia* Fermentation by Triton X-100 for Hypocrellin A Production

**DOI:** 10.3390/ijms21030882

**Published:** 2020-01-30

**Authors:** Xin Ping Li, Yue Wang, Yan Jun Ma, Jian Wen Wang, Li Ping Zheng

**Affiliations:** 1 College of Pharmaceutical Sciences, Soochow University, Suzhou 215123, Chinawangyue_wy@sina.com (Y.W.); jwwang@suda.edu.cn (J.W.W.); 2 Department of Horticultural Sciences, Soochow University, Suzhou 215123, China

**Keywords:** Triton X-100, extractive fermentation, nitric oxide, reactive oxygen species, *Shiraia*, hypocrellin A

## Abstract

*Shiraia* mycelial culture is a promising biotechnological alternative for the production of hypocrellin A (HA), a new photosensitizer for anticancer photodynamic therapy (PDT). The extractive fermentation of intracellular HA in the nonionic surfactant Triton X-100 (TX100) aqueous solution was studied in the present work. The addition of 25 g/L TX100 at 36 h of the fermentation not only enhanced HA exudation to the broth by 15.6-fold, but stimulated HA content in mycelia by 5.1-fold, leading to the higher production 206.2 mg/L, a 5.4-fold of the control on day 9. After the induced cell membrane permeabilization by TX100 addition, a rapid generation of nitric oxide (NO) and hydrogen peroxide (H_2_O_2_) was observed. The increase of NO level was suppressed by the scavenger vitamin C (VC) of reactive oxygen species (ROS), whereas the induced H_2_O_2_ production could not be prevented by the NO scavenger 2-(4-carboxyphenyl)-4,4,5,5-tetramethylimidazoline-1-oxyl-3-oxide (PTIO), suggesting that NO production may occur downstream of ROS in the extractive fermentation. Both NO and H_2_O_2_ were proved to be involved in the expressions of HA biosynthetic genes (*Mono*, *PKS* and *Omef*) and HA production. NO was found to be able to up-regulate the expression of transporter genes (*MFS* and *ABC*) for HA exudation. Our results indicated the integrated role of NO and ROS in the extractive fermentation and provided a practical biotechnological process for HA production.

## 1. Introduction

Hypocrellin A (HA) is a perylenequinone pigment extracted from the fruiting bodies of a pathogenic fungus *Shiraia bambusicola* in bamboos. HA has been widely used in photodynamic therapy (PDT) for skin diseases and is becoming a novel non-porphyrin photosensitizer for the treatment of cancers and viruses [1,2]. Due to the limitations of the wild fungal fruiting bodies and complexity of total chemical synthesis of HA [3], *Shiraia* mycelium culture has become a biotechnological alternative for HA production [4]. Since lower HA yield is the bottleneck of biotechnological production of HA in *Shiraia* fermentation, many process strategies have been applied to *Shiraia* cultures, including medium optimization, treatment of fungal elicitor [5,6], ultrasound stimulation [7] and light radiation [8,9]. Liu et al. (2016) managed to mutagenize *Shiraia* spores using cobalt-60 gamma irradiation to obtain mutated strains for higher HA production [10]. Apart from these conventional optimization methods, extractive fermentation in water-organic solvent two-phase system, also known as perstractive fermentation or milking process, is becoming an efficient strategy to enhance fungal products [11]. In extractive fermentation, organic surfactant is added to permeabilize cells for intracellular products across the cell membrane and extract the fungal products consecutively in the surfactant micelle aqueous solution. Another two-phase system is formed when a nonionic surfactant micelle aqueous solution is at above a certain temperature (cloud point). The cloud point system has a surfactant micelle aqueous solution and a coacervate phase (surfactant-rich phase), which has been extensively studied for the extraction, separation and purification of metal chelates, organic compounds and biomaterials [12]. Recently, nonionic surfactant Triton X-100 (TX100) has been applied successfully as an effective extractant on the perstraction of intracellular pigments produced by *Monascus* [13] and *Talaromyces* [14], the conversion of benzaldehyde into *L*-phenylacetylcarbinol by *Saccharomyces cerevisiae* [15] and microbial transformation of cholesterol by *Mycobacterium* sp. NRRL B-3683 [16]. In the mycelium culture of *Shiraia* sp. SUPERH168, TX100 at 0.2–1.0% (*w*/*v*) was used to induce hypocrellin biosynthesis [17]. We previously screened more surfactants including TX100, Tween-40, -80, sodium dodecyl sulfate (SDS), Brij 52, Span 80 and Pluronic F68, F-127 for the induction of HA in mycelium cultures of *S. bambusicola* and TX100 exhibited significant elicitation on HA production [18]. However, the application of the concept of nonionic surfactant micelle aqueous solution or cloud point system to submerged *Shiraia* fermentation has not been studied.

Great efforts have been made on the selection of different surfactant, optimization of addition time, effects of surfactant concentration, solubility and bioavailability, and fermentation mode in nonionic surfactant micelle aqueous solution [11]. However, the underlying mechanism on the effects of nonionic surfactant on the production of fungal metabolites is still largely unknown. Some factors, including changes in fungal morphology and pellet formation [19], an increase in cell membrane permeability [13], solubilizing the extracellular pigments in micelle aqueous solution [20] and a perstraction effect of surfactant micelles [14] have been suggested as possible action mechanisms of surfactants. In our previous study on *S. bambusicola*, TX100 elicited HA biosynthesis and induced reactive oxygen species (ROS) generation in the mycelia [18]. Since TX100 and other surfactants could incur toxic effects via disruption of cellular membrane [21], it is not surprising that the defense responses to oxidative stress could be initiated by the surfactant exposure. Now, compelling evidence indicates that, in addition to ROS generation, nitric oxide (NO) is an essential biological messenger in plant and mammalian cells under the oxidative stress [22,23]. However, there has been, so far, few reports regarding the regulation of NO on fungal metabolism, and no reports regarding physiological responses, especially on NO and ROS signaling during surfactant exposure in mycelium cultures. Therefore, as a follow-up exploration on stimulating HA production in the fermentation under elicited conditions including the treatments of fungal elicitor [24], the sonication of low-energy ultrasound [7,25], and the radiation of ultraviolet-B [26] or red light [9], we wish to explore the physiological role of NO and ROS in mycelium cultures of *S. bambusicola* in nonionic surfactant micelle aqueous solution. This study may help us understand the mechanism or the signaling regulation in fungal extractive fermentation and provide a novel process strategy for HA production in *Shiraia* fermentation.

## 2. Results

### 2.1. Extractive Fermentation in Micelle Aqueous Solution

**Figure 1:**
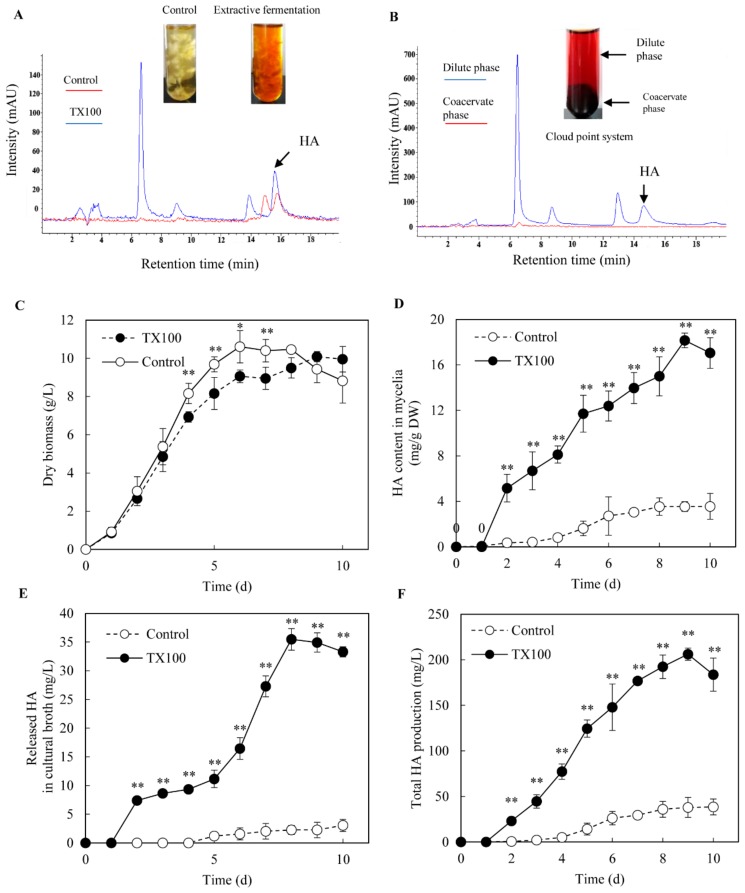
The extractive fermentation of *Shiraia* sp. S9 in nonionic surfactant micelle aqueous solution. (**A**) The chromatogram of HA in *Shiraia* mycelia in the extractive fermentation. (**B**) The chromatogram of HA in cloud point system. TX100 was added at (25 g/L) at 36 h of 8-day fermentation. The supernatant was heated at 75 °C for 1 h and separated into a cloud point system including TX100-rich phase (coacervate phase) and the dilute phase. The time-course of fungal biomass (**C**), HA content in mycelia (**D**), the released HA in cultural broth (**E**) and the total HA production (**F**) in the extractive fermentation. Data shown is the mean ± SD (n=3). Asterisks represent significant differences when compared to control group, *p < 0.05, **p < 0.01.

The biocompatibility, permeability and elicitation effects of nonionic surfactant TX100 to *Shiraia* cells are confirmed in our previous report [18]. Hence, extractive fermentation in submerged culture of *Shiraia* sp. S9 was conducted by adding TX100 at 25 g/L after 36 h of the initial culture. The red perylenequinone pigments of *Shiraia* were majorly accumulated intracellularly in the control culture without TX100 addition, but exported into the broth by extractive fermentation in the nonionic surfactant micelle aqueous solution (Figure 1A). Then, the extracellular broth after 8 days of the extractive fermentation was further subjected to cloud point extraction (Figure 1B). After phase separation in cloud point system at 75 °C, the extracellular pigments were separated into the dilute phase and coacervate phase (TX100-rich phase) whereas HA partitioned mainly to the coacervate phase (Figure 1B). As shown in Figure 1D, HA was an intracellular product as the amount of HA released from cells to medium was less than 8% in the control culture. Although TX100 led to a slight drop (less than 15%) of the mycelium biomass after day 4 (Figure 1C), HA contents in both mycelia and medium were enhanced significantly in the extractive fermentation (Figure 1D,E). The extracellular HA production was increased sharply after TX100 addition to the highest value (35.5 mg/L) on day 8, about a 15.6-fold increase over the control (Figure 1E); whereas the intracellular HA contents were increased by 5.1-fold (Figure 1D). After 9 days of the extractive fermentation, total HA production reached a highest value 206.2 mg/L (Figure 1F), about 5.4-fold of the control.

### 2.2. Effect of TX100 on Fungal Membrane Permeabilization

**Figure 2:**
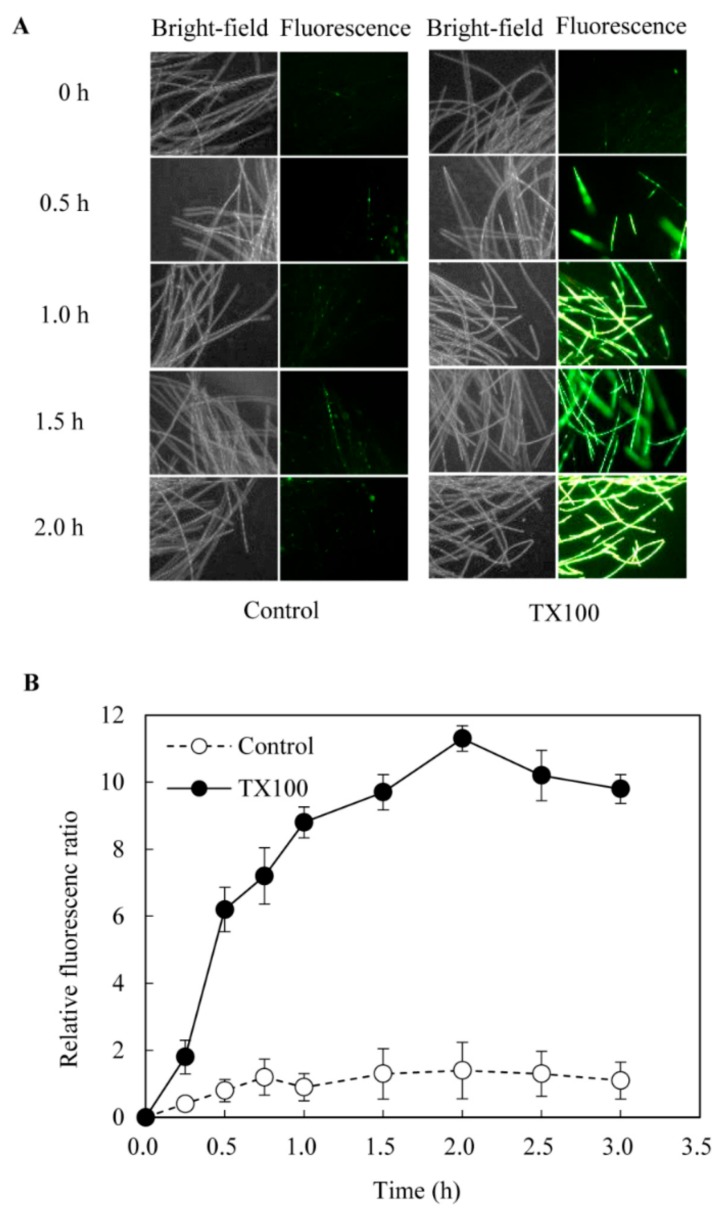
The membrane permeability of hyphal cells in the extractive fermentation (400 ×). (**A**) The membrane integrity of hyphal cells was detected by a nucleic acid stain fluorescent dye SYTOX Green at 0.5 μmol/L. (**B**) The time-course of membrane permeability in the extractive fermentation. The relative fluorescence ratio is defined to the ratio of fluorescence intensity at a given time to that at 0 min. TX100 was added at 25 g/L at 36 h of the fermentation. Data shown is the mean ± SD (n=3).

When TX100 was added to *Shiraia* hyphal cells, the uptake of the high-affinity nucleic acid stain fluorescent dye SYTOX Green was rapidly increased and the fluorescent signal emitted by hyphal cells was clear and strong (Figure 2A), indicating a higher permeability of the cell membrane. The relative fluorescent intensity showed about 8-fold increase after TX100 addition and peaked at about 2 h (Figure 2B), which proved that TX100 could improve the permeability of the fungal cell membrane in the extractive fermentation.

### 2.3. NO Generation in the Extractive Fermentation

**Figure 3:**
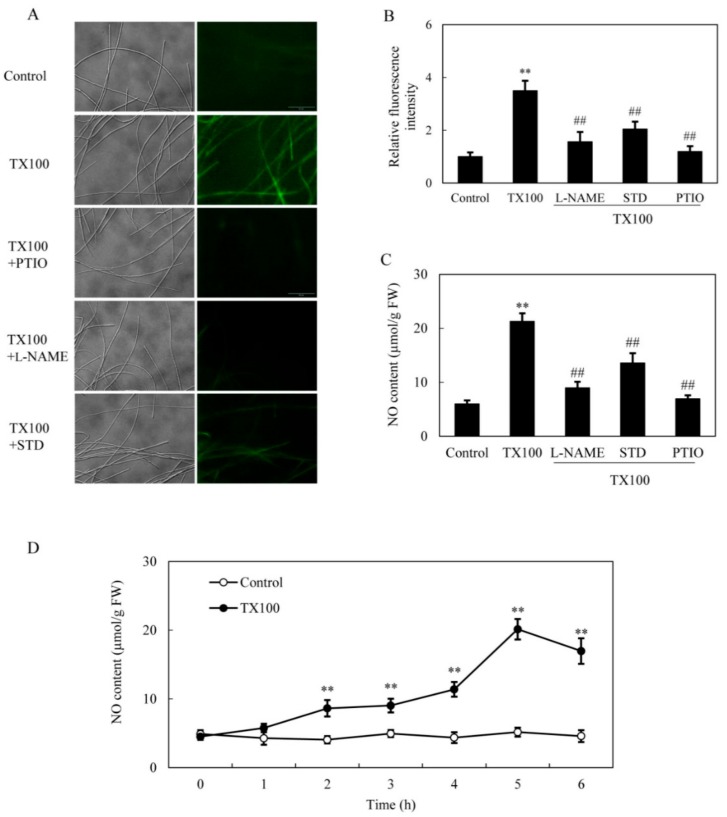
NO generation in the extractive fermentation. (**A**) Bright-field image (left) and fluorescence microscopy of DAF-2 DA-stained mycelium (right) in cultures. TX100 was added at 25 g/L at 36 h of the fermentation. PTIO (100 μM), L-NAME (100 μM) or STD (100 μM) was added 30 min prior to TX100 treatment respectively. The photo was taken after 5 h of TX100 treatment. NO accumulation (relative fluorescence intensity) (**B**) and NO content (**C**) in mycelium after TX100 treatment. (**D**) Time-course of NO contents in mycelium after TX100 treatment. Values are mean ± SD from three independent experiments (^**^p < 0.01 vs. control, ^##^*p* < 0.01 vs. TX100 group).

The induced NO production in *S. bambusicola* mycelia was observed directly on the green fluorescence of 4, 5-diaminofluorescein diacetate (DAF-2 DA)-stained mycelia after 5 h of TX100 addition (Figure 3A, B). The induced fluorescence intensity was suppressed by NO scavengers 2-(4-carboxyphenyl)-4,4,5,5-tetramethylimidazoline-1-oxyl-3-oxide (PTIO), indicating that endogenous NO was response for the increased fluorescence. The induced NO content in mycelium was inhibited by 58% and 36%, respectively, by nitric oxide synthase (NOS) inhibitor *N*_ω_-nitro-l-arginine methyl ester (L-NAME) and nitrate reductase (NR) inhibitor sodium tungstate dihydrate (STD) (Figure 3A–C), suggesting that both NO synthase and NR were the possible sources for NO production. The induced NO accumulated with time and peaked around 5 h, about 3.9-fold over that of the control (Figure 3D).

### 2.4. ROS Production in the Extractive Fermentation

**Figure 4:**
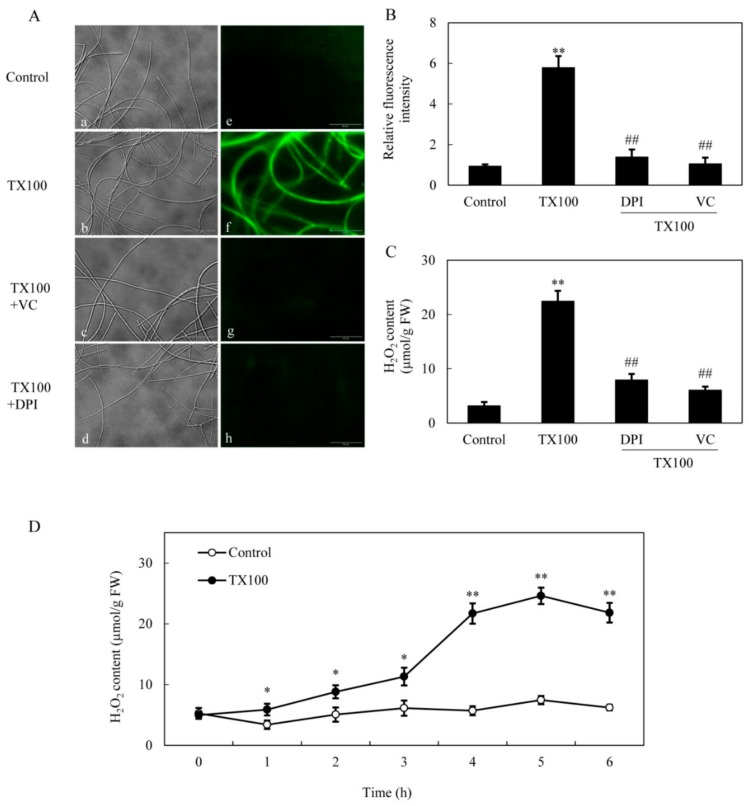
ROS generation in the extractive fermentation. (**A**) Bright-field image (left) and fluorescence microscopy of DCFH-DA-stained mycelium (right) in cultures. TX100 was added at 25 g/L at 36 h of the fermentation. DPI (5 μM) or VC (10 μM) was added 30 min prior to TX100 treatment respectively. The photo was taken after 5 h of TX100 treatment. ROS accumulation (relative fluorescence intensity) (**B**) and H_2_O_2_ content (**C**) in mycelium after TX100 treatment. (**D**) Time-course of H_2_O_2_ contents in mycelium after TX100 treatment. Values are mean ± SD from three independent experiments (^**^p < 0.01 vs. control, ^##^*p* < 0.01 vs. TX100 group).

**Figure 5:**
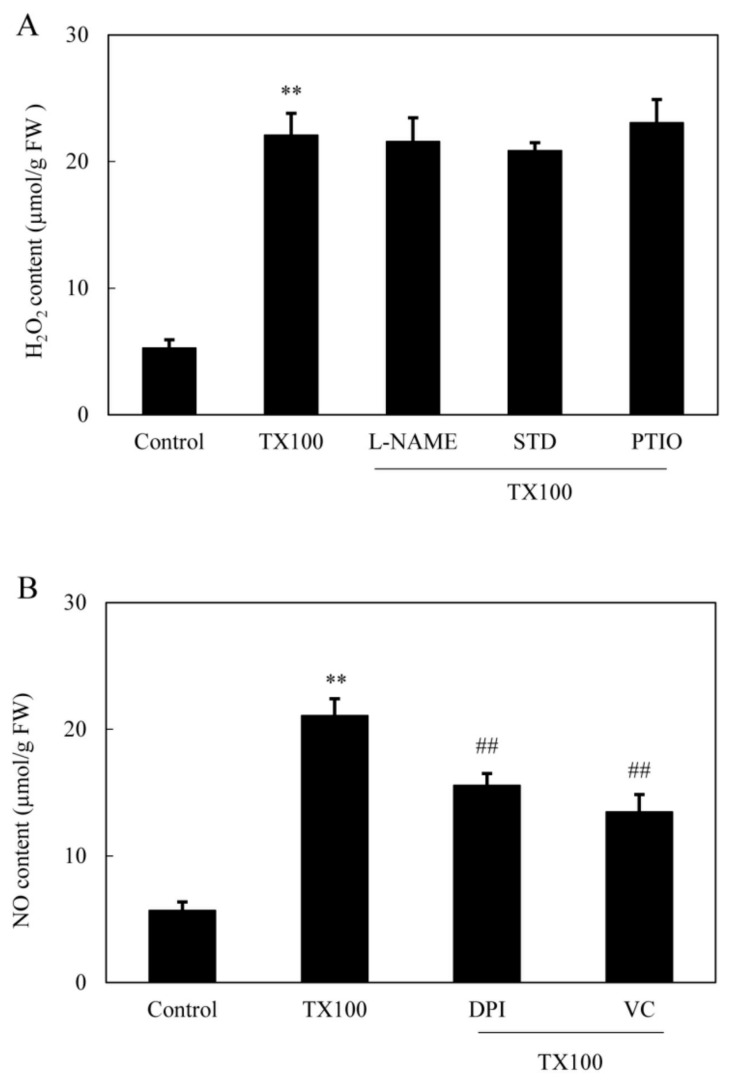
The induced H_2_O_2_ (**A**) and NO (**B**) production in the extractive fermentation and the influence of the donor, inhibitors and scavengers of NO and ROS (same procedure and dosage as specified in Figure 3 and Figure 4). Values are mean ± SD from three independent experiments (^**^p < 0.01 vs. control, ^##^*p* < 0.01 vs. TX100 group).

Simultaneously, TX100-induced ROS production in mycelia was directly observed using the green fluorescent probe of 2, 7-dichlorodihydro fluorescein diacetate (DCFH-DA) (Figure 4A). After 5 h of TX100 treatment, the relative intensity of DCFH-DA fluorescence increased by 6.2-fold over that of control (Figure 4B). The fluorescent increase was blocked strongly by NADPH oxidase (NOX) inhibitor diphenylene iodonium (DPI) and ROS scavenger vitamin C (VC), suggesting that the fluorescence increase in the mycelia was mainly from ROS production by NOX. Moreover, the induced H_2_O_2_ content in mycelia was suppressed significantly by 65%, 73% by DPI and VC, respectively (Figure 4C). The extractive fermentation induced rapid accumulation of H_2_O_2_ around 5 h, reaching a broad peak of 24.6 μmol/g FW (Figure 4D). Although no significant suppression on H_2_O_2_ production was detected after the addition of NO inhibitors including PTIO, L-NAME and STD (Figure 5A), the induced NO production was suppressed by 26% and 36%, respectively, by DPI and VC (Figure 5B), suggesting the relationship between NO and the oxidative stress of the extractive fermentation.

### 2.5. Effect of NO and ROS on HA Production

**Figure 6:**
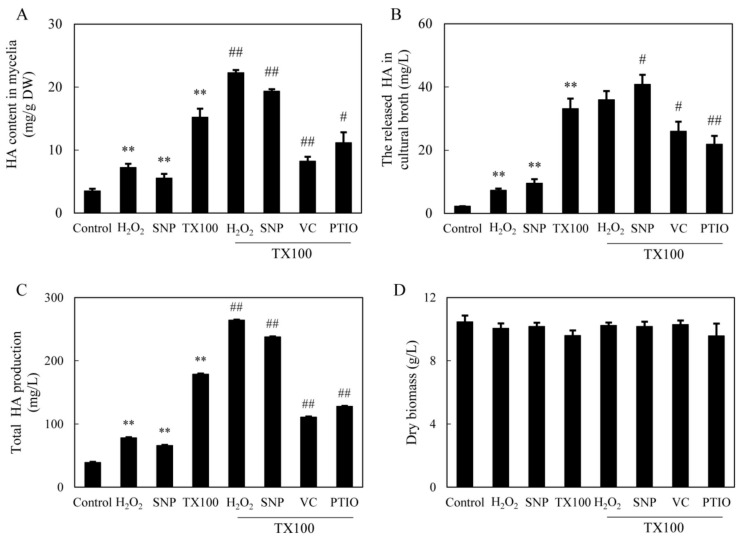
Effects of TX100 on HA content in mycelia (**A**), the released HA in medium (**B**) and total HA production (**C**) and mycelium biomass (**D**), and the influences of the donor, inhibitors and scavengers (same dosage as specified in Figure 3 and Figure 4) of NO and ROS in the extractive fermentation. TX100 was added at 25 g/L at 36 h of the 8-day fermentation. Values are mean ± SD from three independent experiments (^**^p < 0.01 vs. control; ^#^*p* < 0.05 and ^##^*p* < 0.01 vs. TX100 group).

Although the deprivation of ROS and NO in the extractive fermentation did not cause the significant changes in membrane permeability (TX100+VC or TX100+PTIO in Figure S1), HA production in medium was inhibited markedly when ROS and NO generation was inhibited by VC and PTIO (TX100+VC or TX100+PTIO vs. TX100 in Figure 6A–C). Contrarily, the exogenous applied H_2_O_2_ and sodium nitroprusside (SNP) at 0.1 mM alone caused enhancement of intracellular and extracellular HA in the control culture without TX100 (Figure 6A, B). After the application of H_2_O_2_ in the extractive fermentation with TX100, HA content in the mycelia was increased significantly (TX100+H_2_O_2_ vs. TX100 in Figure 6A), whereas both intracellular and extracellular HA was stimulated by the addition of SNP (TX100+SNP vs. TX100 in Figure 6A,B). The addition of H_2_O_2_ or SNP in the extractive fermentation resulted in 7.8-, and 7.0-fold increase of HA production respectively, leading to total HA production 264.1 mg/L and 237.6 mg/L, respectively (Figure 6C). However, the mycelial biomass was not altered significantly by above treatments (Figure 6D). These results indicated the relationship between the induced signals (NO and ROS) and the total HA production (extracellular and intracellular) in the extractive fermentation.

### 2.6. Effect of NO and ROS on Gene Expression for HA Biosynthesis

**Figure 7:**
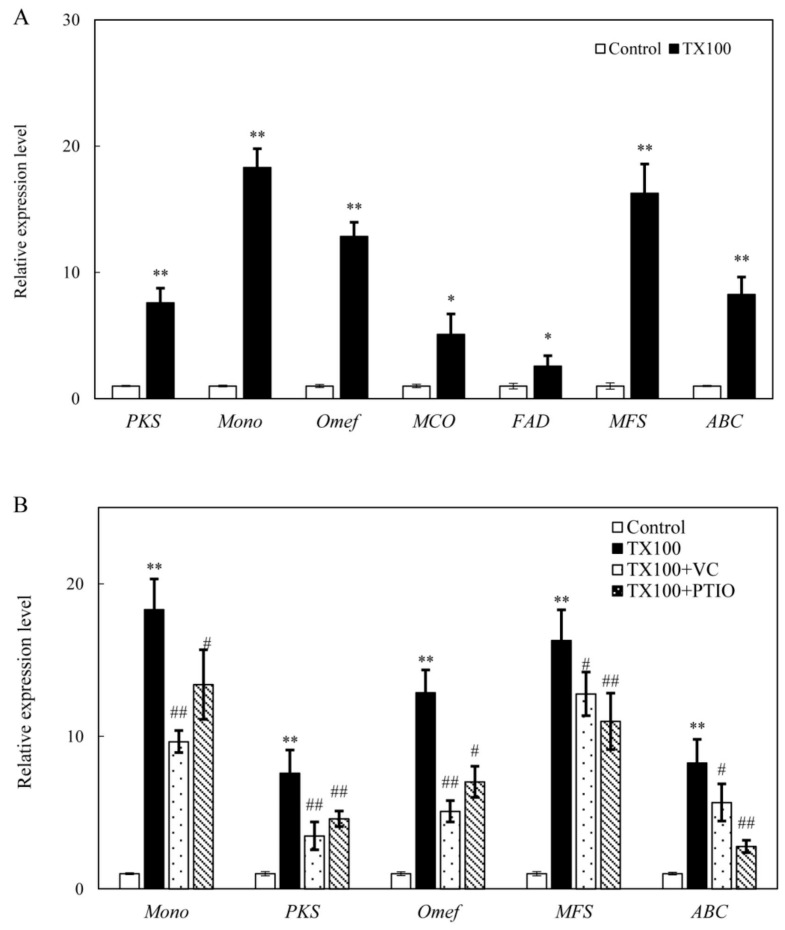
Effect of TX100 on the gene expressions for *Shiraia* HA biosynthesis (**A**) and the influences of the donor, inhibitors and scavengers (the dosage as specified in Figure 3 and Figure 4) of NO and ROS on the gene expressions in the mycelium (**B**). TX100 was added at 25 g/L on day 1.5 for 24 h of TX100 treatment. Values are mean ± SD from three independent experiments (^**^p < 0.01 vs. control; ^#^*p* < 0.05 and ^##^*p* < 0.01 vs. TX100 group).

The expressions of some HA biosynthetic genes were significantly up-regulated after 24 h of the extractive fermentation (Figure 7). TX100 up-regulated gene expressions of polyketide synthase (*PKS*, 7.6-fold), monooxygenase (*Mono*, 18.3-fold), *O*-methyl-transferase (*Omef*, 12.9-fold), multicopper oxidase (*MCO*, 5.1-fold), FAD/FMN-containing dehydrogenase (*FAD*, 2.6-fold), major facilitator superfamily (*MFS*, 16.3-fold) and ATP-binding cassette (*ABC*, 8.3-fold) (Figure 7A). Furthermore, TX100-induced up-regulation of gene expressions could be abolished by VC and PTIO (Figure 7B). The induced expressions of biosynthetic genes including *Mono*, *PKS* and *Omef* were significantly decreased by VC, indicating the main involvement of ROS in HA biosynthesis. Simultaneously, the induced expressions of transporter genes (*MFS* and *ABC*) were repressed significantly after PTIO treatment (Figure 7B), suggesting the possible involvement of NO in the HA release in the medium.

## 3. Discussion

The extent of enhancement of productivity and final product concentration of microbial metabolites is closely associated with the type of extractants and their interaction with the microbial cells in an extractive fermentation process [11]. Due to the benefit of using TX100 as elicitor in *Shiraia* cultures [17,18], we chose TX100 as the addictive to explore the mechanism of extractive fermentation. TX100, as a biocompatible nonionic surfactant has been applied in the perstractive fermentation of *Monascus* fungi to enhance their pigment production, since it has capabilities to alter the permeability of cell membrane for pigment exudation [13], as well as to solubilize the extracellular pigments in micelle aqueous solution [20]. In our previous report [18], TX100 could greatly alter the composition of membrane lipids and increase the ratio of unsaturated/saturated fatty acids of *S. bambusicola* mycelia. In this study, the improved permeability occurred after TX100 addition in the extractive fermentation (Figure 2), making the cell membranes more conducive to the export of intracellular HA. The intracellular HA was secreted into its fermentation broth (Figure 1E) and consecutively extracted into the nonaqueous micelle phase (Figure 1A). Since HA was confirmed to be self-toxic to the fungus in our recent paper [27], the transferring of HA into its nonionic surfactant micelle aqueous solution in extractive fermentation may alleviate self-toxicity of HA on fungal growth or the feedback inhibition of HA biosynthesis. Our results also demonstrated that increase of HA production in the extractive fermentation was from not only HA in the cultural broth, but also the stimulated biosynthesis of HA in mycelial cells (Figure 1). In this study, a higher production of HA (206.2 mg/L) was induced in the extractive fermentation, suggesting a new method for biotechnological process to improve HA yield, and this is also the first report on the induced generation of NO and ROS signals in the extractive fermentation (Figure 3 and Figure 4).

The previous studies have shown that NO was involved in elicited production of polyphenols in submerged cultures of *Inonotus obliquus* by pathogenic *Alternaria alternate* and *Phellinus morii* [28,29], and ganoderic triterpenoid production in the submerged cultures of *Ganoderma lucidum* [30]. Recently, similar reports on effects of induced NO by biotic elicitors from live *Phoma* sp. BZJ6 [31], *Aspergillum niger* [32] and *Phytophthora boehmeriae* [6] were made on *S. bambusicola* for hypocrellin and laccase production. Their studies suggested NO may be one of necessary signal molecules involved in metabolite production in *S. bambusicola* under the elicitation. In present study, the induced NO generation and its involvement in the biosynthesis of HA were verified in the extractive fermentation (Figure 3). The kinetics of TX100-induced NO production is similar to that in *S. bambusicola* cultures with fungal elicitors from *A. niger* [32] and *Phoma* sp. BZJ6 [31], suggesting NO is an early defense response of *S. bambusicola* to the stress. In our experiments, a shorter induction time (around 5 h) for the highest NO production was observed (Figure 3D), while it took about 7.5 h or 2 d for *Shiraia* cultures treated by fungal elicitors to achieve the highest NO production [31,32]. This discrepancy was likely due to different responses to abiotic and biotic elicitors. Although the source of NO in fungi remains obscure, NOS-like proteins have been identified in *Neurospora crassa* [33], *Magnaporthe oryzae* [34], and *I. obliquus* [29]. In our experiments, TX100-induced NO production in *Shiraia* mycelia was inhibited by both the NOS inhibitor L-NAME and NR inhibitor STD (Figure 3), suggesting the possible occurrence of a NOS-like enzyme and NR-dependent side reaction for the induced NO production [35].

TX100 and other surfactants disrupt cellular membrane and induce defense responses [21]. ROS generation including H_2_O_2_ production is one of the earliest fungal defense reactions. The induced ROS generation in *Shiraia* was further verified in our extractive fermentation (Figure 4). The suppression of TX100-induced NO production by DPI (NADPH oxidase inhibitor) and VC (ROS scavenger) suggested that ROS generation may play a regulation role in NO biosynthesis (Figure 5). However, the induced H_2_O_2_ production was not attenuated by NO inhibitors (L-NAME and STD) and scavenger PTIO. Although the accumulation of NO or H_2_O_2_ reached a plateau almost simultaneously around 5 h, the results suggested that NO production may occur downstream of ROS in *S. bambusicola* after TX100 treatment. This is in agreement with the finding from guard cells of *Pisum sativum* exposed to chitosan elicitor that NO inhibitors prevented the chitosan-induced NO levels but did not suppress the ROS production [36]. Recently, we found that ROS generation was one of early signals for the abiotic elicitation on *Shiraia* mycelium cultures for HA production, including the treatments of a low intensity ultrasound [7], a light/dark shift [37] and lanthanum (La^3+^) [38]. Therefore, the existence of interplays between NO and ROS might have been involved in HA biosynthesis in the extractive fermentation.

Previous researches on the transcriptomic profiles and the draft genome sequence of *S. bambusicola* [39,40] provided the putative biosynthetic genes for HA biosynthesis, including *PKS* for the catalyzing of the condensation of acetyl-CoA and malonyl-CoA subunits, *Mono* and *FAD* for polyketide oxidations, and *Omef* for methyl modification on the HA backbone. In the extractive fermentation, TX100 up-regulated expressions of all of these HA biosynthetic genes (Figure 7). It is worth noting that the expressions of *ABC* and *MFS* were increased significantly, while both genes were reported to be involved in HA efflux [38,41]. Accordingly, the addition of TX100 not only enhanced HA contents in mycelia, but also stimulated HA exudation to the medium (Figure 1). The results from our present study have also shown that TX100-induced HA contents in mycelia was enhanced further by NO donor SNP and H_2_O_2_ but suppressed by NO scavenger PTIO and ROS scavenger VC (Figure 6C), whereas the released HA in medium was increased by SNP and deprived by PTIO and VC (TX100+SNP/PTIO/VC vs. TX100 in Figure 6B). These results demonstrated that HA production was regulated by endogenous NO and H_2_O_2_ in the extractive fermentation. The suppression of TX100-enhanced expressions of HA biosynthetic genes (*Mono*, *PKS* and *Omef*) and transporter genes (*MFS* and *ABC*) by NO scavenger PTIO and ROS scavenger VC (Figure 7) provided further support for the involvement of NO and ROS in HA biosynthesis. On the other hand, the combination of SNP/H_2_O_2_ in the extractive fermentation enhanced the HA production much more significantly than the two used separately (TX100 vs. TX100+ SNP/H_2_O_2_ in Figure 6). From biotechnological viewpoint, the combination of the exogenous donors (SNP and H_2_O_2_) of these signal molecules (NO or ROS) in the extractive fermentation is of great practical value to stimulate fungal production in mycelium cultures.

## 4. Materials and Methods

### 4.1. Strains and Culture Conditions

The HA-yielding strain *Shiraia* sp. S9 was isolated from bamboo (*Brachystachyum densiorum*) twigs [42] and registered in China General Microbiological Culture Collection Center as No. CGMCC 16369. The strain was initially incubated on a potato dextrose agar (PDA) medium in a petri dish at 28 °C for 6 day, as previously reported [42]. The subsequent experiments were carried out in shake-flask cultures on a rotary shaker at 200 rpm and at 28 °C for an overall culture period of 8-10 days. For extractive fermentation in nonionic surfactant micelle aqueous solution, specific amount of nonionic surfactant TX100 (Sigma-Aldrich, St. Louis, MO, USA) at 25 g/L was applied to the shake-flask cultures after 36 h of the initial culture according to our previous study [18].

### 4.2. Membrane Permeabilization Assay

Fungal membrane permeabilization was observed using the fluorescence dye SYTOX Green, a high-affinity nucleic acid stain fluorescent dye [43]. After 36 h of the initial culture, fungal mycelium was incubated in the extractive medium with 0.5 μM SYTOX Green, the fluorescence was analyzed by live cell imaging using an Olympus Cell’R IX81 fluorescence microscope (Center Valley, PA, USA) with excitation wavelength of 488 nm and emission wavelength of 538 nm. The relative fluorescence value is defined to the ratio of fluorescence intensity at a given time to that at 0 min.

### 4.3. Detection of NO and ROS Generation

For the detection of NO generation in fungal mycelia, the NO-specific fluorescent probe DAF-2 DA (Sigma-Aldrich, St. Louis, MO, USA) was used [44]. After 36 h of mycelium culture, DAF-2 DA was added to the culture at 30 min prior to TX100 treatment. The fluorescence was observed using an Olympus Cell’R IX81 fluorescence microscope (Center Valley, PA, USA) with excitation/emission wavelengths (470 nm/525 nm). NO contents in mycelia were measured by Griess assay using a Total Nitric Oxide Assay Kit (Beyotime, Jiangsu, China). On the other hand, ROS accumulation in the mycelia was detected using DCFH-DA (Sigma-Aldrich, St. Louis, MO, USA) under an Olympus Cell’R IX81 fluorescence microscope (Center Valley, PA, USA) with excitation/emission wavelengths (485 nm/528 nm). The content of hydrogen peroxide (H_2_O_2_) in mycelium was determined as previously described by Sun et al. (2018) [37]. The mycelia (0.5 g, fresh weight, FW) were ground into homogenate with 5 mL of 0.05 M PBS buffer (pH 7.8) in ice bath and then centrifuged at 12,000 × *g* for 20 min at 4 °C. The supernatant was added to 750 mL of phosphate buffer (pH 7.0) and 1.5 mL 1 M KI. The absorbance was read by a Shimadzu UV-2600 spectrophotometer (Kyoto, Japan) at 390 nm.

### 4.4. Inhibition Experiments on NO and ROS Effects

To study the effects of NO signal during the extractive fermentation by TX100, SNP and PTIO were used as NO donor and scavenger, respectively. L-NAME and STD were used, respectively, as a NOS inhibitor and NR inhibitor. The NO donor and inhibitors used in the experiments were chosen based on previous studies regarding roles of NO in fungi [6,29,45]. l-NAME (100 μM), STD (100 μM) and PTIO (100 μM) were added to the culture at 30 min prior to, and SNP (0.1 mM) simultaneously with TX100 treatment. On the other hand, to investigate the role of ROS generation during the extractive fermentation, the exogenous H_2_O_2_ (0.1 mM) was used as ROS donor. NOX inhibitor DPI (5 μM) and a scavenger VC (10 μM) were used to pretreat for 30 min in mycelium cultures before the TX100 addition according to our previous study [38].

### 4.5. Extraction and Quantification of HA

The extractive fermentation in Triton X-100 micelle aqueous solution was carried out for 8 d. The fermentation broth was subjected to centrifugation at 5000 rpm for 10 min to separate the mycelia. The extracellular supernatant was heated (75 °C) until the formation of two-phase cloud point system: a surfactant-rich phase (coacervate phase) and a dilute phase, and until reaching the equilibrium (1 h). To determine extracellular HA, the extracellular broth in the two phases (50 mL) was exhaustively extracted with ethyl acetate (150 mL). The organic extracts were evaporated in vacuo to afford a residue and re-dissolved in methanol for HPLC analysis. The intracellular HA extraction and quantification were based on the method described in our previous report [7]. HPLC analysis was carried out using Agilent 1260 HPLC systems equipped with 250 × 4.6 mm Agilent HC-C18 column. The sample was eluted with a mobile phase (acetonitrile: water at 65: 35, *v*/*v*) at 1 mL/min and monitored at 465 nm. HA was quantified with genuine standard provided by the Chinese National Compound Library (Shanghai, China). Total HA production refers to the sum of intracellular and extracellular HA.

### 4.6. Quantitative Real-time PCR

The primers of target genes for HA biosynthesis and internal reference gene (18S ribosomal RNA) were listed in Table S1. The relative gene expressions were measured using RT-qPCR on the basis of our previous reports [7].

### 4.7. Statistical Analysis

Each group consists of triplicate experiments (ten flasks per replicate). Student’s *t*-test and one-way analysis of variance (ANOVA) with Dunnet’s multiple comparison tests were performed for the results. Data are expressed as mean ± standard deviation (SD). The *p* value < 0.05 was considered statistically significant.

## 5. Conclusions

Currently, the regulation of surfactant addition on fungal metabolites has not been well explored. In this study, we have successfully demonstrated that the extractive fermentation by TX100 could enhance greatly HA production in *Shiraia* cultures, suggesting an effective process strategy to mycelium cultures. The present study clearly showed the enhanced production of HA in the extractive fermentation is strongly dependent on the induced permeabilization and the signals including NO and ROS. Our finding indicates NO and H_2_O_2_ could serve as new signals for the fungal production by microbial fermentation in nonionic surfactant micelle aqueous solution. Both NO and H_2_O_2_ not only participated in the up-regulation of HA biosynthetic genes, but also enhanced transporter genes (*MFS* and *ABC*) by NO for HA exudation. Since the extractive fermentation has been a successful process strategy for enhancing fungal production, the elucidation of signal roles will not only bring about the understanding of the response mechanisms during the process but also effective strategies for fungal production of desired metabolites.

## Supplementary Materials

Supplementary materials can be found at https://www.mdpi.com/1422-0067/21/3/882/s1.

## Abbreviations

ABC	ATP-binding cassette
DAF-2 DA	4, 5-diaminofluorescein diacetate
DCFH-DA	2, 7-dichlorodihydro fluorescein diacetate
DPI	diphenylene iodonium
FAD	FAD/FMN-containing dehydrogenase
FW	fresh weight
H_2_O_2_	hydrogen peroxide
HA	hypocrellin A
L-NAME	*N*_ω_-nitro-L-arginine methyl ester
MCO	multicopper oxidase
MFS	major facilitator superfamily
Mono	monooxygenase
NO	nitric oxide synthase
NOX	NADPH oxidase
NR	nitrate reductase
Omef	*O*-methyl-transferase
PDA	potato dextrose agar
PDT	photodynamic therapy
PKS	polyketide synthase
PTIO	2-(4-carboxyphenyl)-4,4,5,5-tetramethylimidazoline-1-oxyl-3-oxide
ROS	reactive oxygen species
SDS	sodium dodecyl sulfate
SNP	sodium nitroprusside
STD	sodium tungstate dehydrate
TX100	Triton X-100
VC	vitamin C
