# Supplementary material for: Nitric Oxide and Hydrogen Peroxide Signaling in Extractive Shiraia Fermentation by Triton X-100 for Hypocrellin A Production
Source: Int J Mol Sci. 2020 Jan 30;21(3):882. doi: 10.3390/ijms21030882 (PMC7037624; doi:10.3390/ijms21030882)
Supplement: Supplementary file 1 [file ijms-21-00882-s001.zip › Electronic supplementary material 2020-1-8.pdf]

Electronic supplementary material

Nitric Oxide and Hydrogen Peroxide Signaling in Extractive *Shiraia* Fermentation by Triton X-100 for Hypocrellin A

Xin Ping Li [1], Yue Wang [1], Yan Jun Ma [1], Jian Wen Wang [1] and Li Ping Zheng [2,*]

*Correspondence: lpzheng@suda.edu.cn; Tel.: +86-521-65880195

Table S1. Primers used in RT-qPCR. F: forward primer, R: reverse primer.

Gene symbol	Gene name	Sequence
PKS	Polyketide synthase	F:TGCTGAGGTAGCAGTCAAGC<br>R:TTATGCTACGGTCGTCGCTC
Mono	Salicylate 1-monooxygenase	F:TCTCGGGGAATTATGGCACG<br>R:ACAACCGTTCTCGCATCAGT
Omef	O-methyltransferase	F:GAACTACCTGAAGGCACGCT<br>R:GCTCGGAAGGATACTCGCTC
MCO	Multicopper oxidase	F:TATGGCGCTACGAGTGGAC<br>R:ACTCCCTGGCCGATAACGTA
FAD	FAD/FMN-containing dehydrogenase	F:TGTGACCGCCATCACCTTAC<br>R:TTGTCGTATGGGTGGGAAGC
MFS	Major facilitator superfamily	F:TCCCGTAGCCTTGCTTTCTG<br>R:CCGGCTTCTTCTTGACGCTA
ABC	ATP-binding cassette	F:GACTTGAGCCTATCCGCCTC<br>R:AGAGTCGCCTCTGTGATCCT
18S rRNA	18S rRNA gene	F:GAAAGTTAGGGGATCGAAGA<br>R:TAGTCGGCATAGTTTACGGT

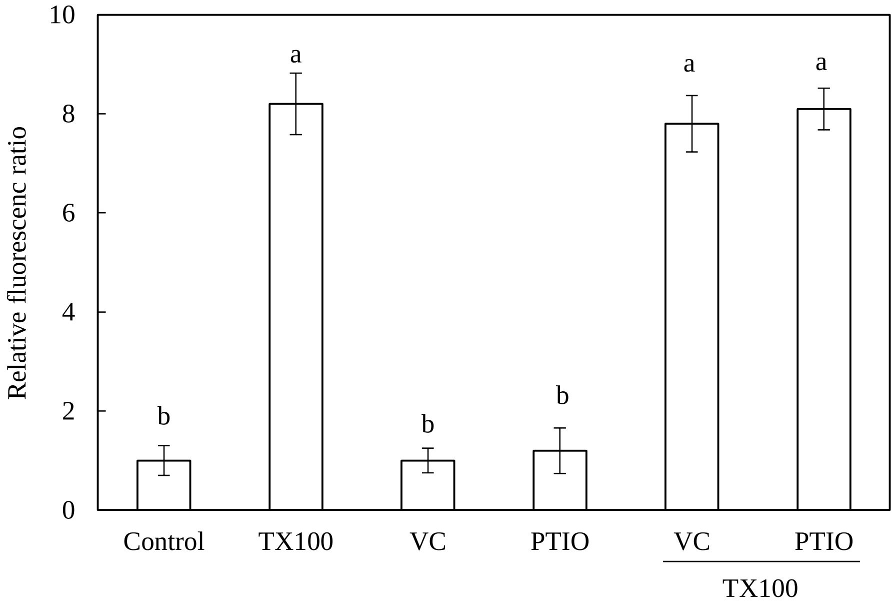

Figure S1. The effect of TX100, VC and PTTIO on membrane permeability of hyphal cells in the extractive fermentation. TX100 was added at 25 g/L at 36 h of the fermentation. The membrane integrity of hyphal cells was detected by a nucleic acid stain fluorescent dye SYTOX Green at 0.5 μmol/L. The dosage and procedure for VC and PTIO addition was specified in Figure 3 and 4. The relative fluorescence ratio is defined to the ratio of fluorescence intensity at a given time to that of the control. Values are mean ± SD from three independent experiments. Different letters above the bars mean significant differences ($p < 0.05$).
